# Supplementary material for: In vivo ephaptic coupling allows memory network formation
Source: Cereb Cortex. 2023 Jul 7;33(17):9877–95. doi: 10.1093/cercor/bhad251 (PMC10472500; doi:10.1093/cercor/bhad251)
Supplement: Supplementary_Material_bhad251 [file supplementary_material_bhad251.pdf]

Supplementary Material

I. Deep neural fields

We modelled data during the delay period of a delayed saccade task. Under a stationarity assumption, we can obtain fixed point network dynamics (Brunel and Wang, 2001). Thus, the variable $V^m(z,t)$ comprises both excitation and inhibition following a mean field description of population activity (Dimitris A. Pinotsis et al., 2013). See also (Pinotsis and Miller, 2022b) for a link to Wilson and Cowan network models. We assume that transmembrane depolarization $\hat{V}^m(z,t) \in \tilde{V}^m$ was sampled from a random process $\tilde{V}^m$ and rewrite Equation (1) by assuming a Taylor expansion

$$\hat{V}^m \approx \sum_{i=0}^{n} w_i H_i \qquad \text{(I.1)}$$

where we linearized $f \circ V^m(z,t) \approx df\, V^m(z,t)$. The connectivity components w_i are found below. The principal axes H_i of the neural field (1) are given by the spatial derivatives of the neural activity (cf. last line in Equation (1) in main text). These contain temporal information, that is, fluctuations around baseline activity at different spatial scales, see (Pinotsis et al., 2017) for details. The name principal axes derives from training the model (2) with a PCA-like algorithm using the cost function (3). For the ephaptic model discussed in main text, the principal axes are given by

$$H_i = \frac{\partial^i (V^m + V_0^e)}{\partial x^i} \qquad \text{(I.2)}$$

Different values of the index i correspond to derivatives of different orders. H_i, i=1,2,3… are called the axes of order i, that is, first, second, third axis etc. Principal axes are matrix-valued functions of dimensionality $N_T x T$, where N_T is the number of trials and T is the

length of the raw LFP time series. We call each entry in these matrices the axis strength. This corresponds to an instantaneous scale factor with which the corresponding component strength must be multiplied to reconstruct the observed LFP. Across-trial averages of principal axes for FEF and SEF were shown in Figures 2 and 3 of (Pinotsis et al., 2017b).

Adding Gaussian noise ε with precision $r^2{}_s = 1/s^2{}_s$, we obtained the following probabilistic model

$$\hat{V}^m \approx \sum_{i=0}^{n} w_0 H_0 + \varepsilon$$
$$\varepsilon \sim (0, s_s{}^2 I) \qquad \text{(I.3)}$$

We then assumed that connectivity components were sampled from a normal distribution $w \sim N(0, I_Q)$. This ensured that they were uncorrelated. Considered mean-centered activity, we obtain Equation (2). We then used a Restricted Maximum-Likelihood (ReML) algorithm to obtain Bayesian optimal values for the connectivity components w_k (Harville, 1977b). This algorithm optimizes the objective function known as Free Energy, *F,* given by Equation (3). This expression of the Free Energy obtains from the sum

$$F = E_{w\sim Q}\left[\log p(Y|w)\right] - D_{KL}\left[Q(w|Y)\|N(0, I_Q)\right] \qquad \text{(I.4)}$$

under a Laplace assumption, where Q is the approximate posterior and after substituting $q \sim Q(w|Z, s_s^2\Pi^{-1})$ and $Y \sim \mathrm{N}(0, HH^T + s^2{}_s I_P)$, see also (Friston, 2008).

II. Bidomain model of the ensemble electric field

According to the theory of electromagnetism, the discontinuity between extracellular and intracellular potential (see Figure 1C) gives rise to dipole sources with moments (Jackson, 1999)

$$p_a = \nabla^2 V^m / r \qquad \text{(II.1)}$$

Here p_a is the moment of a neural ensemble whose center is at location (x_a, y_a), r is the brain resistivity with $r = 2.2$ Ohm (Rush and Driscoll, 1969) and we have assumed that the number of neurons is large and that each cell is very small compared to the distance at which the LFP electrode is placed. Also, the current density $I^a(x_a, y_a)$ that results from EPSPs and IPSPs is given by

$$I^a(x_a, y_a) = p_a/\Omega \qquad \text{(II.2)}$$

where Ω is the total volume of the ensemble. Neglecting ephaptic interactions $V^m \approx V^e$, and the extracellular electric potential generated by the current density $I^a(x_a, y_a)$ is given by

$$V^e(x_e, y_e, w_e) = (4\pi\sigma^e)^{-1} \int I(x_a, y_a) \nabla(1/R) d\Omega \qquad \text{(II.3)}$$

where σ^e is the conductivity of the extracellular space, and R is the distance between the current source at the point $P'(x_a, a)$ of the neural ensemble and the point (x, y) in the extracellular space where we measure V^e, i.e. the location of the LFP electrode, $R = \sqrt{(x - x_a)^2 + (y - \alpha)^2}$, see Figure 1C. Here, *a* is the radius of the grey cylinder in the figure. According to the bidomain model, Equation (II.3) can be written as (Henriquez, 1993; Roth, 1997)

$$V^e(u^e, v^e, w^e) = -(4\pi\sigma^e / \sigma^i) FT^{-1}[\widehat{V}^m(k) W(k)] \qquad \text{(II.4)}$$

where $\widehat{V}^m(k)$ is the Fourier Transform of the transmembrane potential V^m and FT^{-1} is its

inverse Fourier Transform, that is,

$$\widehat{V}^{m}(k)=\int_{-\infty}^{\infty} V^{m}(\rho)e^{ik\rho}d\rho$$
$$FT^{-1}[\widehat{V}^{m}(k)]=V^{m}(\rho)=\int_{-\infty}^{\infty} \widehat{V}^{m}(k)e^{-ik\rho}dk \tag{II.5}$$

Equation (II.4) is the same as Equation (5) in main text where the function $W(k)$ is defined in the second line.

III. Constituent matrices of matrices M and D appearing in Equation (12)

$$A=\begin{bmatrix} \beta+\tau_{EP}^{-1} & Z & 0 & 0 \\ \delta\widehat{K}_{11}^{1} & \beta+\tau_{NA}^{-1}+\delta\widehat{K}_{11}^{1} & \delta\widehat{K}_{12}^{1} & \delta\widehat{K}_{12}^{1} \\ 0 & 0 & \beta+\tau_{EP}^{-1} & 4Z \\ 0 & \delta\widehat{K}_{21}^{1} & 0 & \beta+\tau_{NA}^{-1}+\delta\widehat{K}_{22}^{1} \end{bmatrix}$$
$$B=\begin{bmatrix} 0 & 0 & 0 & 0 \\ 0 & 0 & 0 & 0 \\ 0 & 0 & 0 & 0 \\ \delta\widehat{K}_{21}^{1} & 0 & \delta\widehat{K}_{22}^{1} & 0 \end{bmatrix}$$
$$C=\begin{bmatrix} 0 & 0 & 0 & 0 \\ 0 & \delta\widehat{W}_{11} & 0 & \delta\widehat{W}_{12} \\ 0 & 0 & 0 & 0 \\ 0 & \delta\widehat{W}_{21} & 0 & \delta\widehat{W}_{22} \end{bmatrix}$$
$$D=\begin{bmatrix} \beta+\tau_{EP}^{-1} & Z & 0 & 0 \\ \delta\widehat{K}_{11}^{2} & \beta+\tau_{NA}^{-1}+\delta\widehat{K}_{11}^{2} & \delta\widehat{K}_{12}^{2} & \delta\widehat{K}_{12}^{2} \\ 0 & 0 & \beta+\tau_{EP}^{-1} & 4Z \\ \delta\widehat{K}_{21}^{2} & \delta\widehat{K}_{21}^{2} & \delta\widehat{K}_{22}^{2} & \beta+\tau_{NA}^{-1}+\delta\widehat{K}_{22}^{2} \end{bmatrix}$$
$$E=\begin{bmatrix} \beta+\tau_{EP}^{-1} & Z \\ \delta\widehat{K}_{11}^{1} & \beta+\tau_{NA}^{-1}+\delta\widehat{K}_{11}^{2} \end{bmatrix}$$
$$L=\begin{bmatrix} 0 & 0 \\ \delta\widehat{K}_{12}^{2} & \delta\widehat{K}_{12}^{2} \end{bmatrix}, G=\begin{bmatrix} 0 & 0 \\ \delta\widehat{K}_{21}^{2} & \delta\widehat{K}_{21}^{2} \end{bmatrix}$$

IV. Derivation of Equation (6)

Below, starting from Equation (5), we derive Equation (6). We first assumed that the LFP electrode is at a large distance compared to the size of the neural ensemble. In other words, the radius *a* of the intracellular fiber (grey) is very small compared to the vertical distance to the location of the LFP electrode, $a << y, \quad a \neq 0.$ This is shown by a squashed grey cylinder in Figure 1D. Then, the distance from the surface of the intracellular fiber is approximately equal to *R* (Clark and Plonsey, 1968; Plonsey, 1974). Applying the convolution theorem after noting that the inverse Fourier transform *(FT^{-1})* of $K_0(|k|y)$ is $½(y^2+x^2)$, Equation (4) can be written as

$$V^e(x,y) = a^2(4\sigma^e R)^{-1}\int \Delta V^m dx$$
$$R = \sqrt{(x-x_a)^2 + (y-a)^2} \qquad \text{(IV.1)}$$

An alternative way to derive this expression is to start from the general expression of EP in electromagnetism for sources distributed across the intracellular fiber (Jackson, 1999)

$$V^e(x,y) = R^{-1}\int g(x,y)dxdy \qquad \text{(IV.2)}$$

and consider the density $g(x,y) = g(x) = \gamma\Delta V^m$, $\gamma = \alpha^2(4\sigma^e)^{-1}$, that describes charges distributed across an electrical fiber as a result of the transmembrane current $1/r_i\Delta V^m$. Also, *dxdy* is the elementary source volume element. To calculate the integral in Equation (IV.1), we expand it in *multipoles* following standard theory of electromagnetism (Jackson, 1999). Multipole expansions use *Legendre polynomials* $P_m = P_m(r,\psi)$ that are often expressed in spherical coordinates (r,ψ), see (Figure 1D). Equation (IV.1) can be rewritten as

$$V^e(r,\psi) = \gamma \int \frac{\Delta V^m}{\sqrt{r^2 + x^2 - 2rx\cos\psi}} dx \tag{IV.3}$$

By expanding the denominator in Equation (IV.3), V^e can be expressed in terms of Legendre polynomials P_m as follows

$$V^e(r,\psi) = \gamma \int \frac{\Delta V^m}{r} \sum_{m=0}^{\infty} \left(\frac{x}{r}\right)^m P_m(\cos\psi) dx \tag{IV.4}$$

The neural ensemble is assumed to occupy a fiber of length *L*. The density *g(x)* is zero outside the patch, that is, for $|x| > L/2$ and the integral in the above expression is defined of the interval [-L/2,L/2]. Then, odd terms in the above series vanish. Legendre polynomials of even order are given by the following expressions (Abramowitz et al., 1988)

$$\begin{aligned} &P_0(\cos\psi) = 1 \\ &P_2(\cos\psi) = 1/2(3\cos^2\psi - 1) \\ &P_4(\cos\psi) = 1/8(35\cos^4\psi - 30\cos^2\psi + 3) \\ &\ldots \end{aligned} \tag{IV.5}$$

We can thus evaluate the integrals of the first few terms in the series appearing in Equation (IV.4). This yields the following multipole expansion for the EP

$$\begin{aligned} &V_S^e(r,\psi) \approx \gamma \Delta V^m \begin{bmatrix} 1/r - L^2/(24r^3)(3\cos^2\psi - 1) + \\ +L^4/(640r^5)(35\cos^4\psi - 30\cos^2\psi + 3) \end{bmatrix} \\ &\gamma = \alpha^2 (4\sigma^e)^{-1} \end{aligned} \tag{IV.6}$$

where the first term corresponds to a monopole and we have neglected terms of order five and above. Equation (IV.6) provides an algebraic expression connecting the resting state EP value V_S^e during memory delay to the second derivative of the transmembrane potential V^m. Without loss of generality, we consider a measurement point vertical to the ensemble and at fixed distance *r>0*, for which *ψ=0* (Figure 1C). This simplifies the numerical expressions in Equation (IV.6), which now reads

$$V_S^e \approx \gamma(1/r - L^2/12r^3 + L^4/80r^5)\Delta V^m \qquad \text{(IV.7)}$$

From trial to trial the remembered stimulus changes. Thus the EP and the corresponding EF also change, see (Pinotsis and Miller, 2022b) for details. Because Equation (IV.7) provides the resting value, it needs to be extended into an evolution equation that describes the relaxation process of the EP. We thus assumed a simple fixed point equation $\dot{V}^e = -\tau_{EP}^{-1}V^e + V_S^e$ where V^e decays to its resting value V_S^e with rate $1/\tau_{EP}$.

$$\begin{aligned} \dot{V}^e &= -\tau_{EP}^{-1}V^e + \gamma(1/r - L^2/12r^3 + L^4/80r^5)\Delta V^m \\ E^e &= -\nabla V^e \end{aligned} \qquad \text{(IV.8)}$$

which is Equation (6).

Supplementary Figures

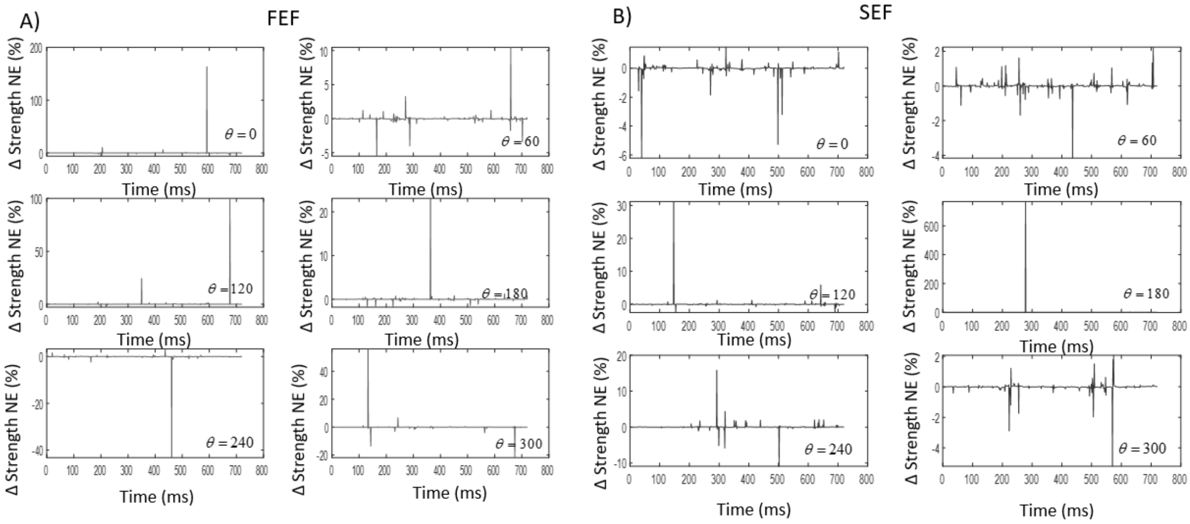

Supplementary Figure 1.

Relative percent changes in endogenous neural activity predicted by the model (principal axis) due to ephaptic coupling for (A) FEF and (B) SEF.

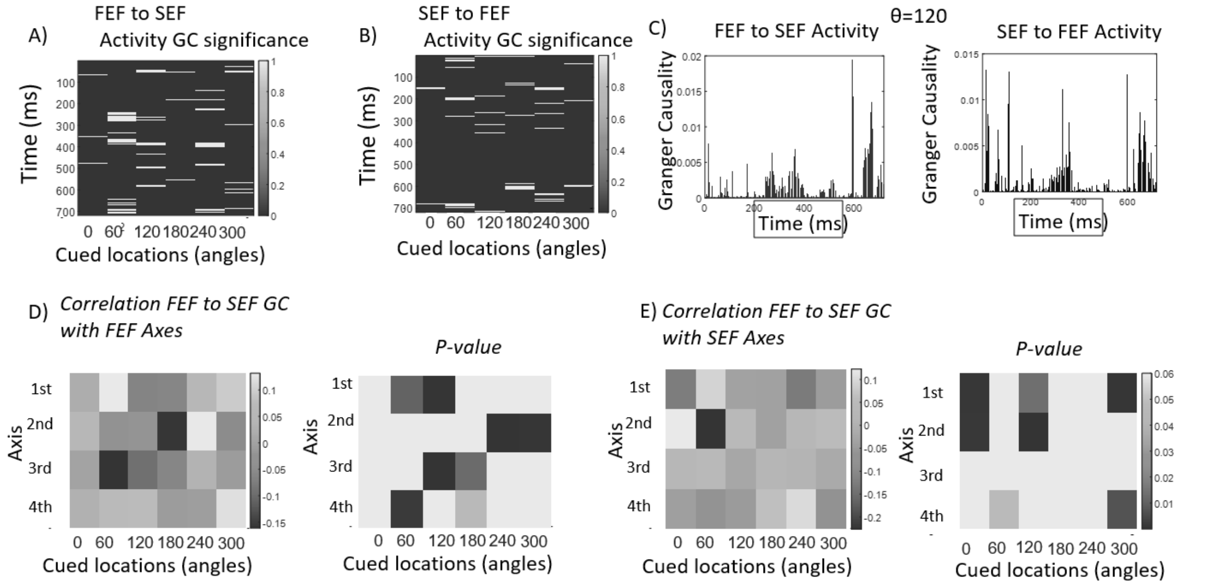

Supplementary Figure 2.

This figure has the same format as Figure 4. The difference is that Granger causality is computed using neural activity, not electric fields.

(A) Time points of significant GC activity interactions from FEF to SEF for all cued locations. Time is shown on the vertical axis and cued locations on the horizontal. Significant interactions are shown in white.

(B) Similar to 4B. Significant GC activity interactions for the reverse direction, from SEF to FEF.

(C) GC strengths (vertical axis) of FEF to SEF (left panel) and SEF to FEF (right panel) activity interactions across time (horizontal axis) for 120 degrees.

(D) Correlations (left panel) and p values (right panel) between FEF principal axes and temporal windows during which GC activity interactions from FEF to SEF were significant. Principal axes are shown on the vertical axis (from first to fourth as we move downwards) and cued locations on the horizontal axis.

(E) Similar to Figure 4D.

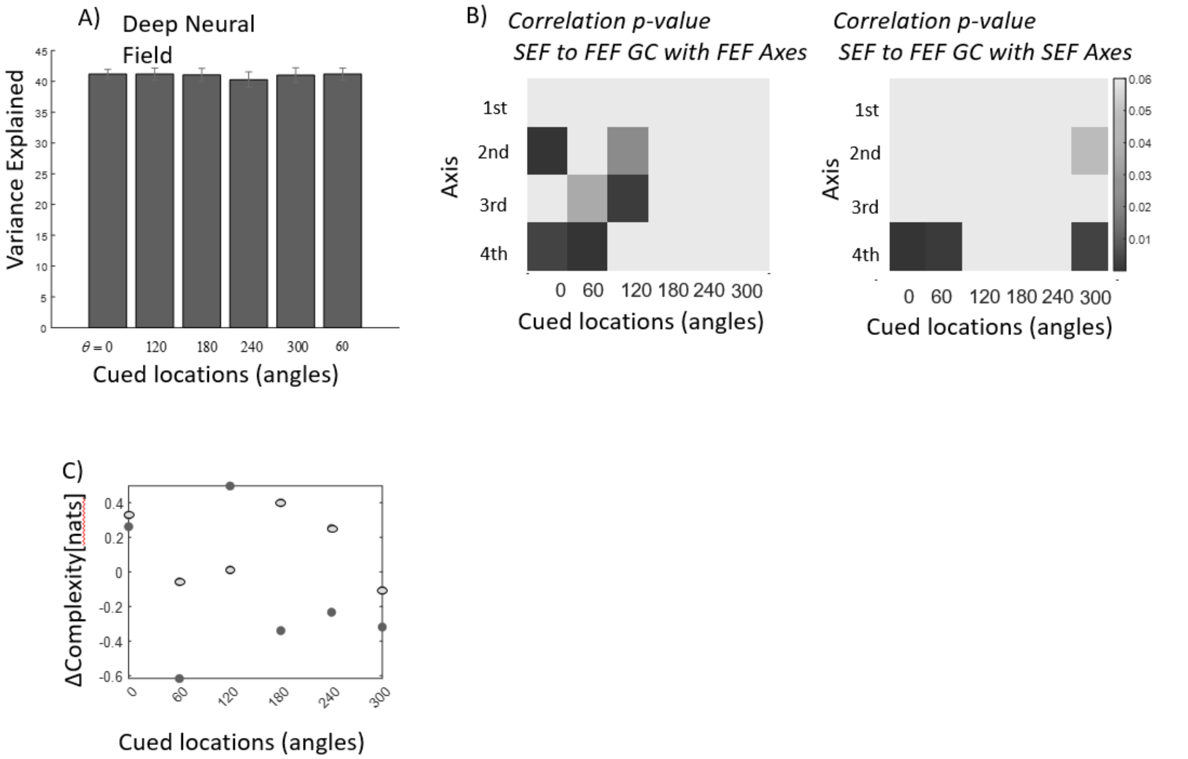

Supplementary Figure 3.

(A) Variance explained by the deep neural field model averaged across trials for each cued location shown on the horizontal axis. Error bars denote standard deviations across trials.

(B) Same as the corresponding right panels in Figures 5D and 5E, but in the reverse direction: SEF to FEF. The plot shows p-values for correlations between axes and temporal windows of significant GC field interactions.

(C) Difference in complexity after fitting the ephaptic and non ephaptic model.The figure follows the format of Figure 2A, where complexity is shown instead of Bayes factors and cued locations are shown on the horizontal axis. FEF differences are shown in grey and SEF differences in black.

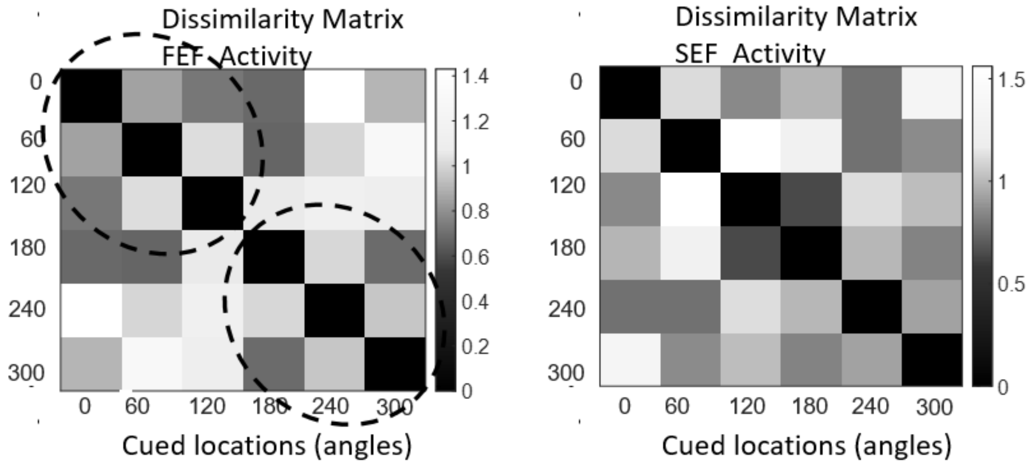

Supplementary Figure 4.

(A) Representation Dissimilarity Matrix (RDM) computed using FEF neural activity.

(B) RDM computed using SEF neural activity.

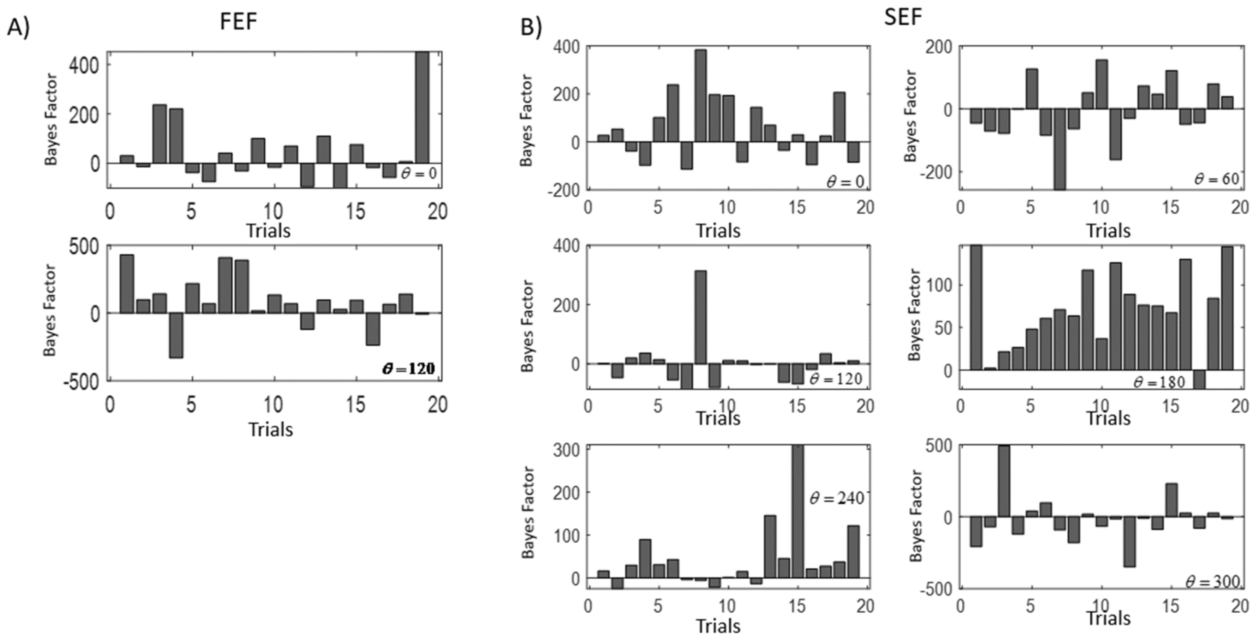

Supplementary Figure 5.

Bayes factor for individual trials and specific cued angles. Different trials are shown on the horizontal axis. The corresponding cued angles are shown at the right side of each plot. (A) Data obtained by fitting the models to FEF data for cued locations at (B) Similar to (A) after fitting the model to SEF data for all cued locations. The ephaptic model fits the data better for most trials.

Supplementary Table

Model parameters

Parameter	*Physiological interpretation*	*Value*
σ^e / σ^i	Ratio of extra-and intra-cellular conductivity	.5
a	Radius of neural ensemble fiber	.2 (mm)
y	Measurement point azimuth	2
τ_X^{-1}	Rate constant of postsynaptic filtering	.25 (1/s)
δ , η	Sigmoid parameters	1,0
